# Digit Ratio (2D:4D) Predicts Self-Reported Measures of General Competitiveness, but Not Behavior in Economic Experiments

**DOI:** 10.3389/fnbeh.2017.00238

**Published:** 2017-12-08

**Authors:** Werner Bönte, Vivien D. Procher, Diemo Urbig, Martin Voracek

**Affiliations:** ^1^Jackstädt Center of Entrepreneurship and Innovation Research, University of Wuppertal, Wuppertal, Germany; ^2^Schumpeter School of Business and Economics, University of Wuppertal, Wuppertal, Germany; ^3^Institute for Development Studies, School of Public and Environmental Affairs, Indiana University, Bloomington, IN, United States; ^4^RWI-Leibniz-Institut für Wirtschaftsforschung, Essen, Germany; ^5^Department of Basic Psychological Research and Research Methods, School of Psychology, University of Vienna, Vienna, Austria

**Keywords:** competitiveness, competition, digit ratio, 2D:4D, prenatal androgen exposure

## Abstract

The ratio of index finger length to ring finger length (2D:4D) is considered to be a putative biomarker of prenatal androgen exposure (PAE), with previous research suggesting that 2D:4D is associated with human behaviors, especially sex-typical behaviors. This study empirically examines the relationship between 2D:4D and individual competitiveness, a behavioral trait that is found to be sexually dimorphic. We employ two related, but distinct, measures of competitiveness, namely behavioral measures obtained from economic experiments and psychometric self-reported measures. Our analyses are based on two independent data sets obtained from surveys and economic experiments with 461 visitors of a shopping mall (Study I) and 617 university students (Study II). The correlation between behavior in the economic experiment and digit ratios of both hands is not statistically significant in either study. In contrast, we find a negative and statistically significant relationship between psychometric self-reported measures of competitiveness and right hand digit ratios (R2D:4D) in both studies. This relationship is especially strong for younger people. Hence, this study provides some robust empirical evidence for a negative association between R2D:4D and self-reported competitiveness. We discuss potential reasons why digit ratio may relate differently to behaviors in specific economics experiments and to self-reported general competitiveness.

## Introduction

Digit ratio (2D:4D), comparing the length of the index finger to the length of the ring finger, is a sexually dimorphic trait with males displaying, on average, a lower digit ratio than females (Manning and Fink, 2008; Hönekopp and Watson, 2010). Since the mid-1990s, digit ratios have attracted research attention because evidence suggests it is related to prenatal androgen exposure (PAE) (Manning et al., 1998; Lutchmaya et al., 2004) and, hence, is often used as a noninvasive retrospective marker for PAE (Ribeiro et al., 2016). Prenatal androgen exposure, with testosterone being the most important androgen, plays an important role in the sexual differentiation of the mammalian brain, which has an enduring influence on behavior (Lombardo et al., 2012; Auyeung et al., 2013; Hines et al., 2015; Manning et al., 2017). These organizational effects of PAE are critically important for the masculinization and sexually differentiated behaviors across the lifespan (Archer, 2006). Those human behaviors that differ by sex are especially expected to be influenced by PAE (Hines et al., 2015).

Individual competitiveness, describing an individual's general tendency to enter competitive situations (Niederle, 2017), is a behavioral trait that is often viewed as sexually dimorphic. Gender differences in individual competitiveness are gaining increasing attention, with behavioral research indicating that women are less willing than men to enter competitions (Croson and Gneezy, 2009; Niederle, 2017). Endorsing the practical relevance of competitiveness, scholars propose that the heterogeneity in sex-specific individual competitiveness may even play an important role for educational and occupational choices (Bönte and Piegeler, 2013; Buser et al., 2014; Flory et al., 2015; Reuben et al., 2015; Bönte et al., 2017b).

This study investigates the association between individual competitiveness and digit ratio (2D:4D). In doing so, we strictly focus on selection into competitive situations and do not examine individual behavior within competitions. While experimental studies on competitiveness focus on gender differences (Croson and Gneezy, 2009), we study within-sex variation of competitiveness and digit ratios. We hypothesize that individuals—men and women—with lower (more masculine) digit ratios are more likely to enter competitive situations than individuals with higher (more feminine) digit ratios.

Links between 2D:4D and other economic behaviors are empirically examined in several studies, with evidence both for and against such links (Millet, 2011; Voracek, 2011). However, to the best of our knowledge, only one study investigates the relationship between selection into competition and 2D:4D (Apicella et al., 2011). In a sample of 93 men aged 18–23, however, Apicella et al. (2011) fail to find a statistically significant correlation between digit ratios of both hands and a behavioral measure of competitiveness obtained from an economic experiment. However, they do not control for risk preferences, even though risk preferences are argued to affect behavior in such economic experiments (Niederle and Vesterlund, 2007) and are also found to be related to 2D:4D (Bönte et al., 2016; Brañas-Garza et al., 2017). These other results suggest that there should be a relationship between 2D:4D and competitiveness in settings as those studied by Apicella et al., especially due to spurious effects by risk preferences. Hence, further tests of this relationship are warranted.

Our study makes several contributions to the literature: First, while existing studies are usually based on single, and rather small, samples, we make use of two large and independent samples, including men and women of different ages, to increase validity of our findings: a general population sample consisting of 461 visitors to a shopping mall (Study I) and a student sample comprising 617 university students (Study II). Second, we employ behavioral measures of competitiveness derived from an experimental design introduced by Niederle and Vesterlund (2007), along with psychometric self-reported measures of competitiveness (Bönte et al., 2017a). A similar approach is used by Brañas-Garza et al. (2017) to examine the relationship between experimental and a simple one-dimensional self-reported measures for risk taking and digit ratio (2D:4D). Going beyond Brañas-Garza et al. (2017), however, we follow Bönte et al. (2017a) and, by employing different psychometric measures of competitiveness, thereby account for the potential multidimensionality of individual competitiveness (Smither and Houston, 1992; Newby and Klein, 2014). Third, in our two studies, we measure digit ratios in different ways. In Study I, an electronic caliper is used to measure 2D:4D, whereas Study II employs a self-reported ruler-based measurement of 2D:4D. This allows for checking the robustness of our results with respect to finger-length measurements. Fourth, we go beyond previous studies and account for two other sex-dimorphic traits viewed as important confounds of competitiveness (Niederle and Vesterlund, 2007) and that are found to be correlated with digit ratio: risk taking (Apicella et al., 2015; Brañas-Garza et al., 2017) and confidence (Da Silva et al., 2015; Neyse et al., 2016). Including these two variables in our regression analyses allows us to check for the robustness of our results and to avoid spurious results due to related confounding effects. Fifth, we discuss the influence of age on the relationship between individual competitiveness and digit ratio, arguing and providing empirical evidence that this relationship is stronger for young people.

The rest of the paper is organized as follows. In section Conceptual Background, we present the conceptual background and discuss the potential relationship between individual competitiveness and digit ratio. In sections Method–Study I and Method–Study II, we describe the methodologies employed in Study I and Study II, respectively. In section Results–Sudies I and II, we present the results of both studies. We further discuss our findings and conclude in section Discussion and Conclusions.

## Conceptual background

### Digit ratio (2D:4D) and prenatal androgen exposure (PAE)

Digit ratio (2D:4D) gained increased interest since Manning et al. (1998) hypothesized that it is related to PAE. Since then, the digit ratio is used in numerous scientific studies as a noninvasive retrospective biological marker for PAE (Ribeiro et al., 2016). More specifically, it is assumed that 2D:4D is negatively correlated with prenatal androgen and positively with prenatal estrogen (Manning et al., 1998, 2017).

The direct link between 2D:4D and prenatal androgen exposure in humans cannot be experimentally demonstrated since ethical constraints ban such experiments. Hence, different attempts are made to provide indirect evidence of the relationship between PAE and 2D:4D. These approaches fall into two groups: correlational studies and experiments with both non-human mammals (Manning et al., 2014) and other vertebrate classes, such as birds (Romano et al., 2005). Correlational studies and quasi-experimental studies are based on three types of evidence (cf., Brañas-Garza et al., 2017): (a) correlation between digit ratio and sex hormones in amniotic fluid; (b) supposed androgen spillovers in zygotic twins; and (c) digit ratios of individuals with sex hormone related syndromes, like Congenital Adrenal Hyperplasia (CAH), Complete Androgen Insensitivity Syndrome (CAIS), and Klinefelter's Syndrome. The results of these studies provide some evidence for the proposed link between PAE and 2D:4D, but results are often mixed and based on small samples (see Manning et al., 2014; Brañas-Garza et al., 2017 for more detailed surveys). The most compelling evidence may come from experiments with non-human mammals that require, however, buying into the assumption that the effects of PAE on human 2D:4D are similar to those observed in experiments with non-human mammals (Manning et al., 2014). The study by Zheng and Cohn (2011), for instance, provides experimental evidence that the 2D:4D ratio is a lifelong signature of prenatal testosterone exposure. Their study shows that, “sexually dimorphic 2D:4D ratios in mice are similar to those of humans and are controlled by the relative levels of androgen and estrogen signaling in utero” (Zheng and Cohn, 2011, p. 16289). In an experiment with rats, Talarovičová et al. (2009) find that an increase in testosterone during pregnancy reduced 2D:4D in both male and female rats by increasing 4D length (i.e., digit ratio becomes more masculinized). Also experimenting with rats, Auger et al. (2013) exposed male rat fetuses to estrogenic and anti-androgenic disruptors, finding that treated rats had more feminized (higher) digit ratios when compared to a control group. Going beyond mammals, Romano et al. (2005) show that a prenatal testosterone treatment affects digit ratios in birds, too. Overall, these findings support the assumption that varying testosterone levels during embryonic life significantly and causally affects digit ratios.

Below we build on the assumption that 2D:4D, in particular the digit ratio of the *right* hand (R2D:4D), is related to PAE, in order to present potential mechanisms for the link between R2D:4D and individual competitiveness^1^. Although the usefulness of digit ratios as a retrospective marker of PAE is challenged in the more recent literature (Hines et al., 2015; Warrington et al., 2016), this assumption neither restricts nor invalidates our empirical analysis since we only examine whether individual competitiveness is related to digit ratio (2D:4D). The fact that the digit ratio is a sexually dimorphic trait shows that it is determined by sex related biological factors, which can be due to prenatal androgen exposure, but also due to other sex-related biological factors; various candidate genes are discussed, for instance, HOX genes^2^. Thus, in our empirical analysis, we choose to take an “agnostic” perspective by focusing on the relationship between digit ratio and measures of individual competitiveness.

### Prenatal androgens, brain development, and sexually differentiated behavior

Embryos are exposed to androgens, estrogens, and other hormones with the resulting balance of sex hormones affecting the nervous system's development. Literature in biology and neuroscience suggests that prenatal androgen exposure has organizing effects on the development of the nervous system and brain in the uterus (Phoenix et al., 1959; Goy and McEwen, 1980; Lombardo et al., 2012; for summaries see Hines, 2010; Auyeung et al., 2013). While the female fetus is exposed to different levels of androgens than the male fetus, there is also considerable variation in prenatal androgen exposure within sexes (Hines, 2010; Auyeung et al., 2013). Previous research suggests that PAE affects behavioral characteristics, such as sexually differentiated childhood behavior in girls and in boys (Auyeung et al., 2009) and some sex-related cognitive, motor, and personality characteristics (Hines, 2010). These organizational effects of PAE on brain development are critically important for the masculinization and sexually differentiated behaviors across the lifespan (Archer, 2006; Hines et al., 2015). Hence, it is expected that, in particular, those behavioral traits showing noticeable gender differences tend to be influenced by PAE and may therefore be correlated with the digit ratio.

### Individual competitiveness and digit ratio

A growing body of literature examines gender differences in individual competitiveness, defined as an individual's general tendency to select into competitive environments (Bönte et al., 2017a)^3^. Reviewing the literature on gender differences in economic experiments, Croson and Gneezy (2009, p. 464) conclude that, “women are more reluctant than men to engage in competitive interactions.” A seminal contribution in this field is the experimental study by Niederle and Vesterlund (2007), who introduce a design for measuring individual competitiveness. This experimental design provides a binary behavioral measure of competitiveness, such that participants have to perform a real effort task and have to choose between a non-competitive piece rate payment scheme and a competitive tournament incentive scheme. Niederle and Vesterlund (2007) find that 73% of the male participants in their experiment selecting themselves into a competitive situation compared to no more than 35% of the females. As performance, risk attitudes, and confidence are themselves subject to gender differences and may also affect the observed choice, Niederle and Vesterlund statistically control for these potential confounds in subsequent regression analyses. They stress that the remaining gender difference points to gender differences in the *preference* for competition. This result is confirmed independently in a number of experimental studies that introduced minor modifications to the original design by Niederle and Vesterlund (see Niederle, 2016 for a survey).

In summary, empirical evidence suggests that individual competitiveness is a sexually dimorphic trait and might, therefore, be related to sex-related biological factors. As mentioned above, masculinization of the human brain in utero due to PAE could result in sexually differentiated behaviors later in life. If 2D:4D is a valid retrospective marker of PTE or PAE, then 2D:4D will tend to be negatively related to more masculine behavioral traits, such as the general tendency to enter competitive situations. Hence, we hypothesize that individuals—men and women—with more masculine (i.e., lower) digit ratios are more competitively inclined than individuals with more feminine (i.e., higher) digit ratios.

### Potentially confounding factors: risk attitudes and confidence

As mentioned above, competitive preferences revealed in economic experiments may not only reflect competitiveness as a specific behavioral trait but they may also reflect other behavioral traits, such as confidence in one's abilities or risk attitudes (Niederle and Vesterlund, 2007). Empirical evidence suggests that women are more risk averse than men both in laboratory experiments and in investment decisions in the field (Croson and Gneezy, 2009). Men also tend to be more (over)confident than women (Lundeberg et al., 1994). Most experimental studies indicate that controlling for risk attitudes and confidence reduces the gender difference in selection into competition, but does not fully eliminate it (Niederle and Vesterlund, 2011; Niederle, 2016).^4^ Moreover, there is some empirical evidence that these two sexually dimorphic confounding variables are correlated with 2D:4D. Several experimental studies investigating the relationship between risk taking and digit ratio provide mixed evidence (Apicella et al., 2015). A more recent study using a large sample (*n* = 704) finds that male and female subjects with lower digit ratios tend to choose riskier lotteries in incentivized experiments, whereas the digit ratio is not associated with self-reported risk attitude (Brañas-Garza et al., 2017). In contrast, Bönte et al. (2016) and Stenstrom et al. (2011) find that digit ratio is negatively associated with self-reported risk attitudes. The empirical evidence is also mixed for the relation between confidence and 2D:4D. Dalton and Ghosal (2014) find that men with lower digit ratios are less likely to set unrealistically high performance expectations. Da Silva et al. (2015) report that low digit-ratio children (preschoolers) show more overconfidence in fine and gross motor skill tasks. Neyse et al. (2016) find that males with low digit ratios are more overconfident about their performance in a non-incentivized treatment, while males with low digit ratios are less overconfident in an incentivized treatment. In view of this evidence, we cannot fully rule out the possibility that individual competitiveness is not directly related to 2D:4D but only indirectly via its association with confidence and risk attitudes. Thus, in our empirical analysis we will control for confidence and risk attitudes, hypothesizing that 2D:4D is independently related to individual competitiveness.

### Age and individual competitiveness

Age might be another factor that affects the relationship between individual competitiveness and 2D:4D. While individual differences and sex differences in 2D:4D already emerge prenatally and digit ratios appear stable over lifetime (Trivers et al., 2006), there are compelling reasons to assume that the association of individuals' general willingness to enter competitive situations and 2D:4D changes across the life span. Individual competitiveness of men and women might be influenced by life experience with respect to education, occupations, and family; in other words, nurture might overwrite nature. Hence, the strength of the association between competitiveness and digit ratio may change because factors other than 2D:4D, like individual experiences, make individuals more or less competitive over the span of life^5^.

Although 2D:4D is stable over lifetime and not associated with adult sex hormone levels (Manning et al., 2004; Hönekopp et al., 2007), hormonal changes across the life span may also influence the relationship between 2D:4D and individual competitiveness. Prenatal testosterone's organizing effects on brain development, in adulthood, moderates the activating effects of current androgen levels (Auyeung et al., 2013; Manning et al., 2014)^6^. Hence, it is likely that the strength of the relationship between 2D:4D and competitiveness depends on individuals' current levels of steroid hormones. Specifically, the relationship between 2D:4D and individual competitiveness—moderated by current testosterone—is expected to be stronger when individuals are young, because men's and women's levels of circulating testosterone gradually decrease with age (Gray et al., 1991; Davison et al., 2005).

To sum up, it is likely that the relationship between 2D:4D and individual competitiveness can be better identified when using samples of young people, because the brain's response to activational steroid hormones decreases with age and because the individual competitiveness of younger people is less likely to be influenced by external factors not related to biology, like experience-based overwriting of individual predispositions (Bönte et al., 2016). Consequently, we hypothesize that individual competitiveness and 2D:4D are more strongly related when using samples of younger people than when using older people.

### Existing evidence and own approach

To the best of our knowledge, the only study examining the relationship between individual competitiveness and digit ratio is Apicella et al. (2011). Based on a sample of 93 men aged 18–23, Apicella et al. (2011) investigate the association between an experimental measure of individuals' preferences to enter competitive situations and four hormonal variables, namely cortisol, circulating testosterone, facial masculinity, and the second-to-fourth digit ratio (2D:4D). Their experimental measure of competitiveness is adapted from Gneezy and Potters (1997): Before conducting a maze solving task, participants are asked to self-select into either a piece rate scheme or a competitive payment scheme (tournament). Apicella et al. (2011) find that the decision to select into a competitive environment is neither significantly correlated with R2D:4D (right hand) nor with L2D:4D (left hand).

Besides the above-mentioned problem that Apicella et al. (2011) do not control for important confound such as risk preferences and confidence, it can also not be ruled out that the relationship between behavioral measures obtained from economic experiments and 2D:4D is influenced by the specific experimental design (context) and, hence, tells us less about an individual's overall competitive disposition. Millet and Dewitte (2009), for instance, demonstrate the relevance of experimental context-specificity for the relationship between economic decision-making and digit ratio. They show that the relationship between 2D:4D and prosocial behavior can turn sign depending on the context, such that the effect might, on average, even disappear.

In order to address the problem that context specificity can alter the relationship between 2D:4D and individual competitiveness, we use two different approaches. First, we use two different real-effort tasks in our two independent studies, respectively. Previous research suggests that different tasks may differently affect the decision to enter competition. For instance, a stronger gender difference in competitiveness is observed if stereotypical male tasks, such as math tasks, are used (Niederle, 2016). Employing different tasks decreases the extent to which our conclusions depend on particularities of a single task. Second, we do not only use behavioral measures of competitiveness, but also self-reported psychometric measures. Following Bönte et al. (2017a), we argue that experimental measures tend to be more context-specific than psychometric scales that are based on general items. The estimated effect of 2D:4D may be stronger if more general measures that are less influenced by a specific context are used (Bönte et al., 2016).

To increase the validity of our research, we employ two independent samples with a total of 1078 individuals, allowing us to have substantial power in each of these samples and to check whether results hold in both samples. We also statistically control for important confounding variables, that is, risk preferences and confidence.

## Method–study I

For Study I, we obtain data from a survey combined with a lab-in-the-field experiment in a shopping mall. Having a general population sample with a large variety in age allows us to investigate the association of 2D:4D and competitiveness conditioned on participants' age.

### Sample and procedures

The survey and lab-in-the-field experiments were conducted in a shopping mall in a large German city for six days in June and October 2014. Visitors were approached and asked whether they would like to participate in a 10–15 min experiment on “decision-making behavior of adults” in return for earnings of at least €5.00. From a total of 488 responses, we exclude 10 due to missing data on finger lengths and 17 due to missing responses to the psychometric measure of competitiveness. In total, 461 responses could be analyzed, including 221 men and 240 women. The average age was 38.26 years (S.D. = 14.37), ranging from 16 to 89 years, with 21 and 58 years marking the tenth and ninetieth percentiles, respectively.

We started with a brief survey on the participant's socio-economic background, e.g., age and gender, which serve as control variables. Moreover, participants assessed two statements concerning their own competitiveness. Next, mall visitors participated in competition games. To create a low-tech environment, the games were conducted with paper and pencil. Further adapting the experimental environment to the time-constrained shopping mall context, we focused on selection into competition under different treatments but not on effects of competition on performance or behavior within competitive environments (cf. Bönte et al., 2017a). Upon completion and just before paying the earnings from the experiment participants were asked to have measured the lengths of the index fingers (2D) and the ring fingers (4D) of both hands in exchange for another €2.00.

### Measurements

#### Behavioral measure of competitiveness

All participants performed a task to collect points and chose the way they were paid for participation. We implemented a math task (cf., Niederle and Vesterlund, 2010) and used an implementation inspired by Mayr et al. (2012). For 30 s, participants verify up to 20 simple single-digit equations (e.g., “7+2+3–6 = 5. Is the result true or false?”). The sets of 20 mathematically equally difficult equations were randomly composed and randomly assigned. One out of two equations was wrong. A correctly verified equation added one point and an incorrect verification subtracted one point. The task description included examples. Before starting with the actual task, participants chose between a non-competitive payment scheme, i.e., a piece-rate of €0.25 for each point of the overall score, and a competitive payment scheme, i.e., €0.50 for each point if the overall score was better than that of a randomly selected previous anonymous participant, €0 otherwise^7^. The behavioral measure of competitiveness is a dummy variable that is zero for participants choosing the non-competitive piece-rate payment and one for participants choosing the competitive payment scheme.

To reduce problems stemming from participants' potential tendency to be self-congruent with respect to their self-reported competitiveness and their plans for their behavior in the experiment, self-reported competitiveness scales were administered *before* participants knew the content of the experiment. Because the experiment is associated with real payoffs, we believe that behavior in the experiment and, hence, the behavioral measure of competitiveness, is less likely to be affected by earlier self-reported competitiveness than vice versa.

#### Psychometric measure of competitiveness

To measure individual competitiveness, we use two items to assess perceived enjoyment associated with competitive situations. The first item (“I like situations in which I compete with others”) is an adaptation of an item from Helmreich and Spence (1978), which is employed in large international surveys run by the European Union, i.e., the Flash Eurobarometer Entrepreneurship 2009 (Bönte and Piegeler, 2013). Replicating the response mode from the Flash Eurobarometer, participants evaluated this item on a 4-point Likert scale from 1 (strongly disagree) to 4 (strongly agree). A second item (“In career terms, I like situations in which I compete with others”) was added to focus more on domains that are of substantial importance to one's professional life. Participants responded on a 7-point scale from 1 (does not apply at all) to 7 (applies strongly). As the scaling of both items varies, we converted the response to the first item to match the range of the second item. The psychometric score for individual competitiveness is the average of these two responses (sample α = 0.77).

#### Digit ratio

At the end of the experiment we asked participants, in exchange for additional money (€2), whether they would allow us to measure the lengths of their ring fingers and the index fingers of both hands. We opted for direct measurement and used an electronic caliper to measure finger lengths^8^.

To distinguish between older and younger participants, we included an indicator that is one if the participant is older than 25 years. This cut-off reflects the 25-percentile (first quartile) of the age distribution. Exploring the effect of 2D:4D for the four age quartiles, we find that there is only a significant effect for the first quartile (see Appendix C).^9^ Hence, and to be consistent with age ranges in our Study II, we chose to focus on the first age quartile.

As important additional control variables, we included risk preferences and confidence. To measure *risk preferences* participants responded to the statement, “In general, I am willing to take risks” on a 7-point scale from 1 (does not apply at all) to 7 (does fully apply). The item is validated by Dohmen et al. (2011), who find that the score of this general risk question is the best all-round predictor of actual risk-taking behavior and is demonstrated to be rather robust (Lönnqvist et al., 2015). In order to create a measure for *confidence*, participants were asked to report how many of 10 potential competitors would have less or an equal number of points; if they were correct they earned another 50 cents. Confidence is measured by subtracting this response from 10 and dividing the resulting score by 10, which approximates the perceived winning probability.

## Method–study II

For Study II, we targeted students in a classroom with a survey and an embedded experiment. This study focuses on a large sample of young people, the group of people we expect to display the strongest association of 2D:4D and individual competitiveness. Going beyond Study I, and exploiting the classroom context, which allows more comprehensive measures, we included an established psychometric scale for individual competitiveness and explore to what extent different dimensions of competitiveness contribute to a correlation between 2D:4D and competitiveness. The behavioral measure of competitiveness is available only for a subsample of all participants. Furthermore, due to the classroom context and the limited time available, we could not rely on experimenters directly measuring participants' digit lengths. Therefore, we employed a self-reported ruler-based measurement of 2D:4D (Bönte et al., 2016).

### Sample and procedures

In winter-terms 2012/13 and 2014/15, we surveyed first- and second-year undergraduate students who attended economics lectures at a German university. At the beginning of the questionnaire, students were informed that their identities were not recorded to ensure confidentiality and that the data would be used solely for scientific purposes. Participants were not informed about the specific nature of the research. From a total of 886 responses, we exclude 77 with missing data of finger lengths, 33 with missing data for self-reported competitiveness, confidence, age, gender, or risk taking. Further, we excluded 86 observations with implausible or inconsistent measures of finger lengths (see below). As we want to focus on young people, we also excluded 72 responses (about 8% of the total sample) from participants older than 25 years. Hence, we employed 618 observations for our analyses. Comparing the restricted (final) and unrestricted sample, we do not find statistically significant differences for our key variables^10^. The majority (82%) of the students were enrolled in business, economics, or related fields such as health economics. The average age was 21.6 years (S.D. = 1.72), ranging from 18 to 25 years, with 20 and 24 years as the tenth and ninetieth percentiles, respectively.

In winter term 2014/2015, we started with a classroom survey, which included questions on self-reported competitiveness, self-efficacy, and risk preferences. There were explicit instructions to wait until all participants had finished this part of the survey. Then participants were provided with a description of an economic experiment. Next all participants chose how they would behave in this experiment. Then participants generated a key that would allow the experimenter to make a random draw of 30 participants who would later participate in the experiment without making public any private information of the participants (like names). Next participants were instructed how to do the measurement of the index, middle, and ring fingers of the right hand and the left hand. After the measurement of the fingers, the participants were asked questions concerning sociodemographic factors, like age and sex. At the end, 30 randomly chosen self-generated keys were listed and these participants performed the experiment and necessary decisions were predetermined based on what they indicated in their survey. The other participants answered questions related to the content of the lecture (economic policy). In winter term 2012/13 the chronology was very similar: first the survey and then the measurement of finger lengths; however, no classroom-experiment was conducted.

### Measurements

#### Behavioral measure of competitiveness

For a subsample of 150 students (in winter-term 2014/15), we obtained a behavioral measure of individual competitiveness derived from a classroom experiment that was embedded into the survey and related confidence measures. Although conducted in class, participation was voluntary. For the experiment, we adopted a design that is frequently used to measure competitiveness (e.g., Niederle and Vesterlund, 2007; Shurchkov, 2012). Participants had to choose between a noncompetitive compensation scheme (“piece-rate”) and a competitive compensation scheme (“tournament”) with respect to their performance in a real task. Specifically, participants had to answer 20 trivia questions on various areas of general knowledge within 5 minutes (questions taken from Eberlein et al., 2011). For each question, participants had to choose the one correct answer out of four given options. Before choosing the payment scheme, all participants received 4 example questions, which they were asked to solve (without any incentives) to familiarize themselves with the task and to gain an impression of the level of difficulty. Students were informed that they could earn up to €20.00 when performing in the task. To save time, however, not all students had to participate in the real task. After the survey, we collected the paperwork with potential participants' decisions and randomly selected 30 of them. The selected students were asked to join the experimenter to perform their task. Questions were presented on a quiz sheet and could be answered in any order. No feedback was provided during the quiz. The payoffs were then paid according to their decisions and the decisions of randomly matched partners. Those participants who previously chose piece-rate, received 50 cents for every correctly answered question in the quiz. The scores of those participants who chose the tournament payment scheme during the survey, were compared to the score of another randomly matched participant^11^. The participant with more correct answers (“the winner”) received 100 cents for every correct answer. The other participant received 0 cents. In case of a tie, the winner was determined randomly. The *behavioral measure of competitiveness* is a dummy variable that is zero for participants choosing the non-competitive piece-rate payment and one for participants choosing the competitive tournament payment.

As in Study I and for the same reasons, self-reported competitiveness scales were administered before participants knew the content of the incentivized behavioral measure of competitiveness.

#### Aggregate psychometric measure of competitiveness

As the first self-reported measure, we employed an adaptation of the competitiveness subscale of the Work and Family Orientation Scale (WOFO; Helmreich and Spence, 1978). This measure aggregates individuals' enjoyment of interpersonal competition but also individuals' desire to do better than others and their desire to win in interpersonal situations (Houston et al., 2002). To stay within a general context easily applicable to the sample of young students, we replaced the item “I enjoy working in situations involving competition with others” with an item that refers to a general rather than a work-specific context: “I like situations in which I compete with others.” The score for this *aggregate measure of competitiveness* is calculated as the average score of responses to the five items of the competitiveness subscale of WOFO (α = 0.77).

#### Enjoyment of competition

Empirical studies using larger sets of items confirm that the scale by Helmreich and Spence (1978) does not reflect a unidimensional concept of competitiveness but comprises different dimensions of competitiveness (Houston et al., 2002; Newby and Klein, 2014). To account for the enjoyment one receives from competition, our second measure of competitiveness focuses on the enjoyment of competition. We included the highest loading item from Newby and Klein's (2014) “general competitiveness” subscale (“*I enjoy competing against others*.”) and the highest loading reverse-coded item from Smither and Houston (1992) emotion factor (“*I find competitive situations unpleasant*”) (see Appendix B). Participants responded to each item on a 7-point scale from “does not apply at all” (1) to “fully applies” (7). The score for enjoyment of competition is calculated as the average scores of both items (α = 0.71).

#### Aggregate competitiveness not driven by enjoyment of competition

To better differentiate between enjoyment of competition and other dimensions of competitiveness that are captured by the aggregate measure of competitiveness, we employed a residualization technique to partition variation in the aggregate measure into two uncorrelated parts (for a similar approach see Bönte et al., 2017a), where one part is not driven by variation in enjoyment of competition. Residualization is implemented by an ordinary least squares regression where the aggregate score of the HS-Scale is the dependent variable and the aggregate score of the EC-Scale is the only explanatory variable. The measure of “competitiveness not driven by enjoyment of competition” is given by the residual plus the constant (RHS = residualized HS-scale).

#### Digit ratio

**Figure 1:**
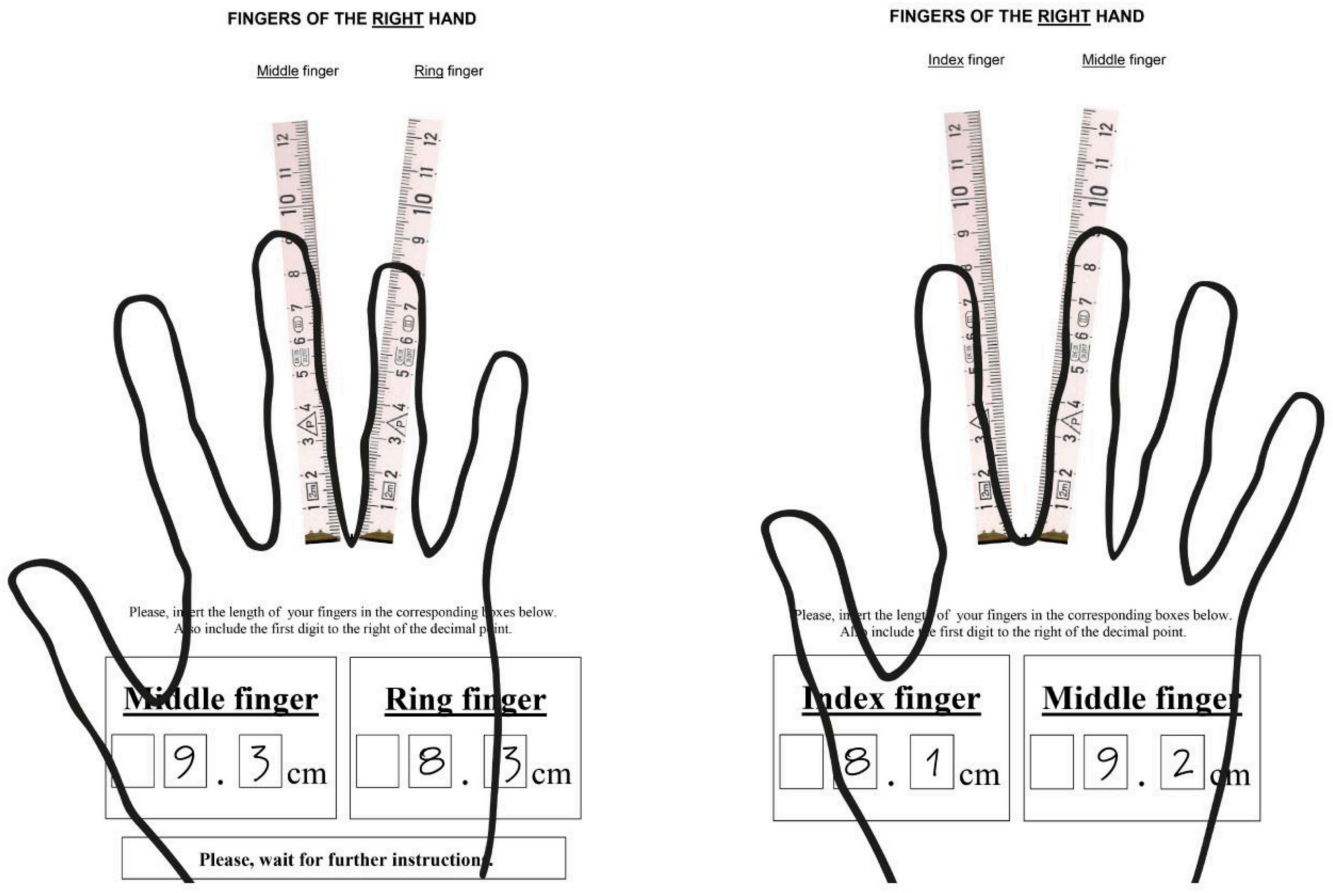
Self-Measurement of finger length.

We employ a self-reported ruler-based measurement of 2D:4D. On four sheets of the questionnaire, two rulers were displayed which were arranged as a triangle, with the rulers starting with zero at the point where they met (see Figure 1). Students marked the length of the ring finger and the length of the middle finger (1st sheet) and then marked the length of the middle finger and length of the index finger (2nd sheet) of the right hand. The same measurement was completed for the left hand (3rd and 4th sheet). Verbal instructions were given on how to do the measurement (e.g., how to position the hand and that the tip of a finger is relevant for measurement, but not the finger nails). We obtained the 2D:4D by dividing the length of the index finger (2D) by the length of the ring finger (4D). Since it is very likely that self-reported measurement of finger length is associated with substantial measurement error, we took measures to detect and drop responses with implausible or unreliable 2D:4D measurements. We extend the measurement approach of Manning and Fink (2008) by exploiting that the middle fingers of both hands are measured twice. We excluded 78 observations where the two measurements of the same middle finger of a hand (once in conjunction with the index and then together with the ring finger) differ by more than 10%, which we interpreted as indicating a substantial lack of reliability for the individually self-measured finger lengths. This is advantageous as the judgment of reliability is based on a finger that does not form the variables of interest. Furthermore, we excluded 8 observations where the 2D:4D did not fall into the usually observed range of 0.8–1.2 (cf., Hönekopp and Watson, 2010; Bönte et al., 2017a; Manning et al., 2017). Visual inspection of the latter observations showed that these outliers tend to be the result of errors when marking the length of fingers on rulers^12^.

In our regression analyses, we control for gender, risk preference, and confidence. Gender is a dummy indicating female participants, risk taking is measured by participants' agreement (from 1—“does not apply at all” to 7—“applies strongly”) with the statement, “In general, I am willing to take risks.” Following Bönte et al. (2017a), we measured confidence in four ways: In contrast to Study I, the data of Study II also contain a measure of general confidence (not related to the experiment), measured by participants' agreement (from 1—“does not apply at all” to 7—applies strongly”) with the statement “Generally, when facing difficult tasks, I am certain that I will accomplish them” (see Bönte and Piegeler, 2013, as an adaptation of an item from Chen et al., 2001). Given that in a specific context, participants may employ different heuristics to form beliefs about their own and others' performances when choosing to select into competitions, we include three distinct measures (cf. Bönte et al., 2017a): We asked participants to forecast their own numbers of correctly answered questions (confidence: own performance) and the average score of all other participants (confidence: average performance). Participants also estimated the percentage of other participants who correctly answered more questions than they themselves do; as in our first study, subtracting this number from 100 and dividing the resulting number by 100 provides an approximation of the estimated winning probability (confidence: winning probability).

## Results–sudies I and II

### Replication of stylized facts related to digit ratio and individual competitiveness

We first explore whether we can replicate the finding of previous research indicating that 2D:4D and individual competitiveness are sexually dimorphic. In both studies (see Tables 1-I, II), we find that female participants display larger 2D:4D and this effect is stronger for the right than for the left hand (Manning and Fink, 2008; Hönekopp and Watson, 2010). Calculating Cohen's d for the difference between sexes is larger for the right hand (I: *d* = 0.19, II: *d* = 0.42) than for the left hand (I: *d* = 0.15, II: *d* = 0.24). While for the general population sample (Study I) the values are lower, the values observed in the student sample (Study II) are not significnatly different from values reported by Hönekopp and Watson (2010) for direct measurements of the right hand (*d* = 0.353, S.E. = 0.040) and left hand (*d* = 0.284, S.E. = 0.044). We further observe in Study I that 2D:4D of the right hand and the left hand do not correlate with age (see Table 1-I).

**Table 1-I:** Summary statistics and correlations (Study I).

**No**.	**Variable**	**Mean**	**S.D**.	***N***	**Pearson correlation coefficients**						
					**1**	**2**	**3**	**4**	**5**	**6**	**7**
**COMPETITIVENESS MEASURES**											
1	Behavioral	0.529	0.500	461	1						
2	Self-reported	4.350	1.534	461	0.234^***^	(0.77)					
**DIGIT RATIOS**											
3	R2D:4D (right-hand)	0.991	0.037	461	−0.030	−0.119^*^	1				
4	L2D:4D (left-hand)	0.989	0.038	461	−0.016	−0.031	0.506^***^	1			
**CONTROL VARIABLES**											
6	Female	0.521	0.500	461	−0.157^***^	−0.238^***^	0.095^*^	0.075	1		
7	Age (>25 years)	0.740	0.439	461	−0.044	−0.061	−0.007	0.001	−0.025	1	
8	Risk taking	4.735	1.428	461	0.108^*^	0.345^***^	−0.038	−0.045	−0.139^***^	−0.030	1
9	Confidence: Winning prob. (0-1)	0.522	0.173	461	0.301^***^	0.109^*^	0.017	−0.004	−0.285	0.048	0.016

*R(L)2D:4D = 2D:4D of right (left) hand. Where available, Cronbach's alpha is reported in parentheses on the diagonal*.

*To explore if the correlations between competitiveness and 2D:4D depend on age, we also report these correlations conditioned on the age dummy. The correlation with the behavioral measure is statistically insignificant for both age groups (≤25: r = −0.014, p = 0.883; >25: is r = −0.036, p = 0.507). As expected, however, we observe that the correlation with the self-reported measure is large for young and smaller and even statistically not significant for the older participants (≤25: r = −0.279, p = 0.002; >25: is r = −0.066, p = 0.223)*.

*Significance levels: ^+^p < 0.10*,

* *p < 0.05*,

** p < 0.01 and

*** *p < 0.001*.

**Table 1-II:** Summary statistics and correlations (Study II).

**No**.	**Variable**	**Mean**	**S.D**.	***N***	**Pearson correlation coefficients**										
					**1**	**2**	**3**	**4**	**5**	**6**	**7**	**8**	**9**	**10**	**11**
**COMPETITIVENESS MEASURES**															
1	Behavioral measure (BM)	0.320	0.468	150	1										
2	Self-reported aggregate (HS)	4.586	1.169	618	0.239^**^	(0.77)									
3	Self-reported enjoyment (EC)	4.453	1.380	618	0.355^***^	0.585^***^	(0.71)								
4	Residualized aggregate (RHS)	2.378	0.948	618	0.026	0.811^***^	0.000	1							
**DIGIT RATIOS**															
5	R2D:4D (right-hand)	0.994	0.053	618	−0.013	−0.101^*^	−0.161^***^	−0.008	1						
6	L2D:4D (left-hand)	0.978	0.056	618	−0.060	−0.115^**^	−0.107^**^	−0.065	0.433^***^	1					
**CONTROL VARIABLES**															
7	Female	0.560	0.497	618	−0.435^***^	−0.257^***^	−0.318^***^	−0.087^*^	0.205^***^	0.116^**^	1				
8	Risk taking	4.635	1.407	618	0.247^**^	0.192^***^	0.267^***^	0.044	−0.101^*^	−0.060	−0.151^***^	1			
9	Conf.: General	4.985	1.277	618	0.192^*^	0.247^***^	0.326^***^	0.070	−0.078^+^	−0.058	−0.169^***^	0.260^***^	1		
10	Conf.: Own perf. (0–20)	10.51	3.521	150	0.361^***^	0.245^**^	0.226^**^	0.124	−0.008	−0.142^+^	−0.325^***^	0.253^**^	0.204^*^	1	
11	Conf.: Average perf. (0–20)	9.300	2.818	150	−0.017	0.042	0.047	0.015	−0.070	−0.074	−0.234^**^	−0.006	0.011	−0.350^***^	1
12	Conf.: Winning prob. (0–1)	0.586	0.186	150	0.222^**^	0.239^**^	0.268^***^	0.087	0.010	−0.109	−0.322^***^	0.179^*^	0.203^*^	0.453^***^	0.092

*R(L)2D:4D = 2D:4D of right (left) hand. Where available, Cronbach's alpha is reported in parentheses on the diagonal*.

Significance levels:

+ *p < 0.10*,

* *p < 0.05*,

** *p < 0.01*,

*** *p < 0.001*.

Our experimental and self-reported measures of individual competitiveness also replicate previous findings related to gender differences (e.g., Croson and Gneezy, 2009). Both the behavioral measures and self-reported psychometric measures of competitiveness are negatively correlated with the female dummy variable, suggesting that men, on average, are more competitively inclined than women (see Tables 1-I, II).

For the general population sample (Study I) and its self-reported competitiveness, the calculated level of Cohen's d (*d* = 0.49) is close to the value reported by Bönte (2015, Table 1) for a representative sample of German citizens (*d* = 0.41). In both our studies, the behavioral measures and the self-reported measures of competitiveness are significantly correlated, suggesting that both types of measures overlap in measuring an individual's tendency to select into competitive situations (see Tables 1-I, II). For Study II, we see that this association is stronger for enjoyment of competition (EC) than for Helmreich and Spence's (1978) aggregate measure of competitiveness (HS) and almost absent for the residualzied measure (RHS) not reflecting the variation related to enjoyment of competition. This suggests that selection into competition is not driven by the desire to win or to perform better in competitions. Our following analyses, thus, focus on the narrower measure of enjoyment of competition rather than Helmreich and Spence's multi-faceted measure.

### Correlational analyses of the relationships between 2D:4D and competitiveness

Both correlation tables (Tables 1-I, II) show that the association of individual competitiveness with 2D:4D is generally stronger for the right hand than for the left hand. This conincides with previous studies suggesting that the right-hand 2D:4D tends to be more strongly affected by prenatal testosterone than the left-hand ratio (Lutchmaya et al., 2004; Hönekopp and Watson, 2010; Zheng and Cohn, 2011) and that significant correlations between sex-dependent behavioral traits and digit ratio are predominantly found for the right hand (Fink et al., 2004; Hampson et al., 2008).

To explore if—as we expect—the correlations between competitiveness and R2D:4D (right hand) depend on age, we also split the sample of the general population into younger (25 years or less) and older (more than 25 years) participants^13^. The correlation with the behavioral measure is not statistically significant for both age groups (≤25: *r* = −0.014, *p* = 0.883; >25: is *r* = −0.036, *p* = 0.507). However, we observe that the correlation with the self-reported measure is larger and statistically significant for younger participants, but smaller and not even statistically significant for older participants (≤25: *r* = −0.279, *p* = 0.002; >25: is *r* = −0.066, *p* = 0.223).

### Basic regression analyses controlling for between sexes variation

Since individual competitiveness (Croson and Gneezy, 2009) and R2D:4D (Hönekopp and Watson, 2010) are sexually dimorphic, we cannot exclude the possibility that the correlation between them is only driven by the sexual dimorphism of these variables and not by variation within sexes. Therefore, we control for participants' sex in our regressions. For Study I with the general population sample, we additionally allow the association between 2D:4D and competitiveness to depend on age. Specifically, we include a dummy variable for participants who are older than 25. In both studies, the relationships between 2D:4D with the behavioral measures were analyzed using logistic regression analyses and the relationships with the self-reported measures were analyzed using ordinary least squared regressions analyses (see Tables 2-I, II).

**Table 2-I:** Basic regression analyses (Study I).

**Model**	**Behavioral measure (logistic regression)**				**Self-reported measure (ordinary least squares regression)**			
	**1**	**2**	**3**	**4**	**5**	**6**	**7**	**8**
R2D:4D	−0.846	0.417			−4.064^*^	−9.862^**^		
	(2.594)	(5.079)			(1.899)	(3.686)		
L2D:4D			−0.252	−4.775			−0.521	−0.813
			(2.523)	(5.268)			(1.859)	(3.794)
Age (>25 years)		1.483		−6.080		−7.996^+^		−0.623
		(5.846)		(5.948)		(4.247)		(4.303)
R2D:4D × Age		−1.726				7.831^+^		
		(5.895)				(4.283)		
L2D:4D × Age				5.915				0.393
				(6.006)				(4.347)
Female	−0.629^***^	−0.637^***^	−0.633^***^	−0.644^***^	−0.702^***^	−0.702^***^	−0.728^***^	−0.733^***^
	(0.190)	(0.191)	(0.190)	(0.191)	(0.139)	(0.139)	(0.140)	(0.140)
Constant	1.286	0.207	0.700	5.350	8.743^***^	14.664^***^	5.245^**^	5.710
	(2.565)	(5.032)	(2.492)	(5.218)	(1.878)	(3.652)	(1.836)	(3.754)
Observations	461	461	461	461	461	461	461	461
Fit index LL/*R*^2^	−313.00^**^	−312.40^*^	−313.09^**^	−312.00^**^	0.066^***^	0.077^***^	0.057^***^	0.061^***^
Fit statistic (χ^2^/F)	(11.50)	(12.70)	(11.40)	(13.49)	(16.21)	(9.57)	(13.83)	(7.47)

*R(L)2D:4D = 2D:4D of right (left) hand. Table reports estimated coefficients and standard errors (in parentheses). The effect of R2D:4D on self-reported competitiveness for the older participants (>25 years) is −9.862 +7.831 = −2.031 with S.E. = 2.198 and p = 0.356)*.

Significance levels:

+ *p < 0.10*,

* *p < 0.05*,

** *p < 0.01*,

*** *p < 0.001*.

**Table 2-II:** Basic regression analyses (Study II).

**Model**	**Behavioral measure (logistic regression)**		**Self-reported measure (EC) (ordinary least squares regression)**	
	**1**	**2**	**3**	**4**
R2D:4D	4.207		−2.597^*^	
	(3.749)		(1.010)	
L2D:4D		0.195		−1.754^+^
		(3.542)		(0.950)
Female	−2.091^***^	−1.970^***^	−0.827^***^	−0.860^***^
	(0.412)	(0.394)	(0.108)	(0.107)
Constant	−3.796	0.129	7.497^***^	6.649^***^
	(3.674)	(3.443)	(0.995)	(0.926)
Observations	150	150	618	618
Fit index LL/*R*^2^	−79.24^***^	−79.88^***^	0.111^***^	0.106^***^
Fit statistic (χ^2^/F)	(29.57)	(28.30)	(38.32)	(36.54)

*R(L)2D:4D = 2D:4D of right (left) hand. Table reports estimated coefficients and standard errors (in parentheses)*.

Significance levels:

+ *p < 0.10*,

* *p < 0.05*,

^**^p < 0.01,

*** *p < 0.001*.

In Tables 2-I, II, we observe that being female is rather robustly, and independent of the measure of competitiveness, negatively associated with competitiveness. Our regression analyses consistently demonstrate that the relationships of digit ratios of the right (R2D:4D) and the left (L2D:4D) hand with the behavioral measure of competitiveness are negligibly small and statistically insignificant. However, we consistently observe—across both samples—a negative relationship of the right-hand digit ratio (R2D:4D) with the self-reported measures of competitiveness. For Study I, we observe that this relationship is significantly weaker for the older participants. In fact, calculating the effect for the older participants, we observe that it is statistically not significant (Table 2-I, Model 6: −9.862 + 7.831 = −2.031, S.E. = 2.198, *p* = 0.356).

### Controlling for important confounding effects

In a next step, we go beyond existing research (Apicella et al., 2011) by taking into account and controlling for risk preferences and confidence. Thereby we can rule out that the omission of these important variables creates spurious correlations between self-reported competitiveness and 2D:4D or suppresses correlations between 2D:4D and the behavioral measure of competitiveness. As explained in section 2, individuals' risk preferences and confidences may influence individuals' decisions to select into competition (Niederle and Vesterlund, 2007), with existing research suggesting that 2D:4D is related to individuals' risk preferences (Apicella et al., 2015; Brañas-Garza et al., 2017) as well as individuals' confidence (Da Silva et al., 2015; Neyse et al., 2016). Therefore, we perform regressions where we also include measures for risk preferences and confidences (see Tables 3-I, II, Models 1, 2, 5, and 6).

**Table 3-I:** Regression analyses controlling for important confounding variables (Study I).

**Model**	**Behavioral measure (logistic regression)**				**Self-reported measure (ordinary least squares regression)**			
	**1**	**2**	**3**	**4**	**5**	**6**	**7**	**8**
R2D:4D	0.884		2.236		−9.602^**^		−9.027^*^	
	(5.294)		(5.532)		(3.489)		(3.649)	
L2D:4D		−4.572		−2.087		−1.617		−1.183
		(5.537)		(5.774)		(3.595)		(3.771)
Age (> 25 years)	3.007	−5.686	2.772	−3.161	−7.927^*^	−2.172	−7.132^+^	−0.852
	(6.116)	(6.242)	(6.393)	(6.489)	(4.021)	(4.084)	(4.208)	(4.264)
R2D:4D × Age	−3.301		−3.169		7.790^+^		6.956	
	(6.168)		(6.443)		(4.055)		(4.241)	
L2D:4D × Age		5.483		2.824		1.989		0.628
		(6.306)		(6.561)		(4.127)		(4.312)
Risk taking	0.149^*^	0.153^*^	0.164^*^	0.166^*^	0.339^***^	0.342^***^	0.356^***^	0.357^***^
	(0.071)	(0.071)	(0.074)	(0.074)	(0.046)	(0.047)	(0.048)	(0.049)
Conf.: Wining Prob.	0.376^***^	0.371^***^	0.358^***^	0.354^***^	0.051	0.048	0.027	0.027
	(0.066)	(0.066)	(0.068)	(0.068)	(0.039)	(0.040)	(0.041)	(0.041)
Female	−0.262	−0.272	−0.297	−0.299	−0.518^***^	−0.553^***^	−0.595^***^	−0.629^***^
	(0.207)	(0.207)	(0.218)	(0.217)	(0.138)	(0.139)	(0.145)	(0.146)
Constant	−3.082	2.326	−4.307	−0.014	12.418^***^	4.516	11.949^**^	4.181
	(5.287)	(5.495)	(5.535)	(5.737)	(3.474)	(3.570)	(3.638)	(3.746)
Observations	461	461	418	418	461	461	418	418
Fit index LL/*R*^2^	−292.08^***^	−292.00^***^	−264.81^***^	−264.84^***^	0.177^***^	0.163^***^	0.196^***^	0.182^***^
Fit statistic (χ^2^/F)	(53.34)	(53.51)	(48.47)	(48.42)	(16.29)	(14.69)	(16.66)	(15.27)

*R(L)2D:4D = 2D:4D of right (left) hand. Table reports estimated coefficients and standard errors (in parentheses). Models 3, 4, 7, and 8 exclude those participants who indicated that their dominant hand is the left hand*.

Significance levels:

+ *p < 0.10*,

* *p < 0.05*,

** *p < 0.01*,

*** *p < 0.001*.

**Table 3-II:** Regression analyses controlling for important confounding variables (Study II).

**Model**	**Behavioral measure (logistic regression)**				**Self-reported measure (EC) (ordinary least squares regression)**				
	**1**	**2**	**3**	**4**	**5**	**6**	**7**	**8**	**9**
R2D:4D	3.122		2.625		−1.998^*^		−1.994^*^		−3.580^+^
	(3.845)		(4.165)		(0.958)		(0.978)		(2.069)
L2D:4D		1.296		5.347		−1.352		−1.356	
		(3.928)		(4.428)		(0.898)		(0.937)	
Risk taking	0.156	0.155	0.222	0.229	0.158^***^	0.161^***^	0.172^***^	0.175^***^	0.088
	(0.176)	(0.174)	(0.189)	(0.189)	(0.037)	(0.037)	(0.038)	(0.038)	(0.089)
Conf: General	0.130	0.142	0.169	0.165	0.256^***^	0.257^***^	0.261^***^	0.263^***^	0.173^+^
	(0.178)	(0.176)	(0.189)	(0.187)	(0.041)	(0.041)	(0.041)	(0.041)	(0.094)
Conf: Own perf.	0.180^*^	0.182^*^	0.165^+^	0.177^*^					0.005
	(0.080)	(0.080)	(0.086)	(0.087)					(0.043)
Conf: Average perf.	−0.026	−0.023	−0.089	−0.087					−0.015
	(0.088)	(0.088)	(0.094)	(0.094)					(0.048)
Conf: Winning prob.	−0.310	−0.192	−1.006	−0.909					0.927
	(1.359)	(1.342)	(1.543)	(1.510)					(0.756)
Female	−1.701^***^	−1.612^***^	−1.924^***^	−1.911^***^	−0.662^***^	−0.686^***^	−0.658^***^	−0.686^***^	−0.659^*^
	(0.493)	(0.474)	(0.548)	(0.533)	(0.104)	(0.103)	(0.106)	(0.105)	(0.281)
Constant	−5.924	−4.314	−4.666	−7.506	4.800^***^	4.132^***^	4.721^***^	4.056^***^	6.701^**^
	(4.011)	(4.332)	(4.260)	(4.771)	(0.991)	(0.920)	(1.012)	(0.955)	(2.152)
Observations	150	150	131	131	618	618	581	581	131
Fit index LL/*R*^2^	−73.27^***^	−73.55^***^	−64.42^***^	63.88^***^	0.208^***^	0.206^***^	0.213^***^	0.210^***^	0.193^***^
Fit statistic (χ^2^/F)	(41.52)	(40.97)	(33.98)	(35.05)	(40.32)	(39.66)	(39.03)	(38.38)	(4.20)

*R(L)2D:4D = 2D:4D of right (left) hand. Table reports estimated coefficients and standard errors (in parentheses). Models 3, 4, 7, and 8 exclude those participants who indicated that their dominant hand is the left hand. Model 9 additionally excludes participants for whom the behavioral measure of competitiveness is not available*.

Significance levels:

+ *p < 0.10*,

* *p < 0.05*,

** *p < 0.01*,

*** *p < 0.001*.

We observe rather consistently across the different models that risk taking and confidence affect competitiveness. Risk preferences are positively associated with competitiveness, though it misses statistical significance for the behavioral measure in Study II. With one exception, in each analysis at least one measure of confidence tends to be positively associated with competitiveness. Only in Study I, where we only have a context-specific measure of confidence available, we do not observe a statistically significant association with self-reported competitiveness (see Table 3-I, Models 5 and 6). This lack of a relationship between specific confidence and general competitiveness may result from violations of the compatibility principle suggesting that predictors and criterion should be specified at the same level of specificity (cf. Ajzen and Fishbein, 2005; Bönte et al., 2017a). Observing that in Study II, the context-specific measures are not, but the general confidence measure is, related to the general self-reported measure of competitiveness supports this reasoning.

Regarding our main explanatory variables, we still do not observe relationships of 2D:4D with the behavioral measure of competitiveness; hence, the confounding effects do not suppress relationships of 2D:4D with behavioral measures of competitiveness. For self-reported measures of competitiveness, we observe that relationships with 2D:4D remain robust for the right hand. For Study II, the relationships with right-hand and left-hand 2D:4D become smaller and, for the left-hand, it does not even reach conventional levels of statistical significance.

As the hand preference displays interactions with effects of 2D:4D (Manning and Peters, 2009), our estimations may be biased, possibly underestimating the effect of 2D:4D. Hence, we complement our analyses with estimations excluding those participants who indicated having a preference for the left-hand (Tables 3-I, II, Models 3, 4, 7, and 8). Our results do not change substantially. While previously not significant effects of 2D:4D on behavioral competitiveness still do not reach any meaningful level of statistical significance, previously significant effects on self-reported competitiveness remain statistically significant.

While the comparison between behavioral and self-reported measures of competitiveness are based on the same sample in Study I, in Study II, the behavioral measure is only available for a subsample of those for whom we have the behavioral measure available. Differences in statistical significance may, hence, result from sample differences. As an additional robustness check, we therefore also estimated the effect on the self-reported measure on the same subsample (see Table 3-II, Model 9 compared with Model 3). We see that the significant results still hold, although on a substantially weaker level; hence, the difference we observe between behavioral and self-reported measures of competitiveness—as in Study I—should not be attributed to sample differences and, particularly, not to the smaller samples size.

As a last more exploratory analysis, we acknowledge that the effects of digit ratios might be gender-specific, such that the relationships differ for men and women. Our estimations testing the gender differences based on an interaction with a gender contrast code, which are reported in Appendix A, however, do not point to gender differences.

## Discussion and conclusions

To investigate the association between individual competitiveness and digit ratio (2D:4D), this study employs two independent samples with a total of 1078 individuals. While Study I is based on a general population sample (461 visitors at a shopping mall), Study II is based on a student sample (618 students at a university). We use these two independent samples to replicate and validate our findings. Moreover, individual competitiveness is measured in two different ways: by behavioral measures obtained from incentivized behavioral experiments and by self-reported psychometric measures.

The results of both studies suggest that the associations between behavioral measures of competitiveness and digit ratios are not statistically significant. This confirms, using a much larger sample and including men and women, the finding reported by Apicella et al. (2011) for a small sample of 93 young men. Moreover, although we use two different real effort tasks in the incentivized experiments in Study I (math task) and in Study II (quiz task), the results are not affected by these task differences.

In contrast to our results regarding the behavioral measure, we find a negative and statistically significant relationship between psychometric measures and 2D:4D in both studies. Our specific findings suggest that psychometric scales reflecting enjoyment of competition are significantly related to the right-hand digit ratio (R2D:4D). The results remain robust when applying slightly different psychometrics scales reflecting individuals' perceived enjoyment of competition. In Study II, we additionally used a seven-item scale introduced by Helmreich and Spence (1978) that also reflects individuals' desire to perform better than others and their desire to win in interpersonal competitions (Houston et al., 2002). Following Bönte et al. (2017a), we employ a residualization technique to identify the part of the HS-scale that is not driven by variations in enjoyment of competition. Our estimation results show that R2D:4D is not significantly correlated with the residual part that reflects variations in the desire to perform better and to win against others. Hence, our results imply that the digit ratio is, first and foremost, related to enjoyment of competition, suggesting that individuals with low (more masculine) digit ratios tend to select into competition not primarily for winning a competition but for the sake of competition itself.

Previous research shows that statistically significant associations between sex-dependent behavioral traits and digit ratio are predominantly found for the right hand (Fink et al., 2004; Hampson et al., 2008). Our observation that the left-hand digit ratio is either not or more weakly associated with competitiveness than the right-hand digit ratio confirms this finding. Our theoretical consideration indicate that it is important to additionally control for potentially confounding variables, namely individuals' confidence and risk attitudes (Niederle and Vesterlund, 2007), which tend to be related to both digit ratio (2D:4D) and selection into competition. Our results show that while the estimated effect is robust for the right-hand digit ratio (R2D:4D) in both studies, it is not for the left-hand digit ratio (L2D:4D). More specifically, the estimated coefficient is still statistically significant for R2D:4D even when controlling for individuals' confidence and risk attitudes. In contrast, the estimated coefficient of L2D:4D becomes statistically insignificant in Study II. This result provides further evidence that sex-dependent behaviors, like individual competitiveness, are predominantly associated with the right-hand digit ratio (R2D:4D).

Moreover, our exploratory analyses indicate that the strength of the relationship between digit ratio and individual competitiveness tends to depend on age. Based on a general population sample, we find that the relationship between individual competiveness and the right-hand digit ratio (R2D:4D) is stronger for younger people (age ≤25). This might be explained by the fact that competitive preferences of younger people are less likely to be influenced by external factors not related to digit ratios (e.g., experiences in education, jobs, and family). Moreover, the relationship between individual competitiveness and the digit ratio may be stronger for young people because the average level of circulating testosterone is higher in younger people, males (Gray et al., 1991) and females (Davison et al., 2005) and the strength of this relationship might be positively moderated by the level of circulating testosterone (van Honk et al., 2012). Hence, future research might consider that the effects of digit ratio (2D:4D) on individual competitiveness and other sexually dimorphic behaviors are moderated by both age and, possibly, circulating testosterone.

Our finding that the digit ratio (R2D:4D) is associated with the self-reported psychometric measures of competitiveness but not with the behavioral measures deserves a more detailed discussion. On the one hand, a significant association between R2D:4D and self-reported enjoyment of competition might be spurious due to confounding effects related to self-reported measures. While we already go beyond previous studies by controling for risk taking and confidence as the most important confounding variables, there might be other more subtle confounding effects. If participants, despite anonymization, want to display specific characteristics, then the significant association might indicate that individuals with low R2D:4D want to display enjoyment with competition. While this could theoretically be the case, controling for risk taking and confidence and not identifying a related effect for the HS-scale, which includes an individual's declared wish to perform better than others and their willingness to win, any potentially confounding effect must be rather specific to self-reported enjoyment of competition.

On the other hand, and as a more substantive explanation for the asymmetric effect, one could argue that in economic experiments, participants have to make decisions in very specific experimental settings and empirical evidence suggests that, for instance, variation in the type of real effort tasks influences an individual's decision to select into competition (Niederle, 2016). Moreover, the results reported by Millet and Dewitte (2009) show that context in experiments can affect the relationship between behavior in experiments and the digit ratio. Although employing two different real effort tasks, performing a classroom and a lab-in-the-field experiment, and make use of a student and a general population sample, the finding of both an insignificant relation between the digit ratio and behavioral measures as well as a significant relationship between the digit ratio and self-reported measures of competitiveness is robust with respect to different contexts and samples.

Our finding that the digit ratio is significantly correlated with the self-reported measures of competitiveness but not with the behavioral measures does not imply, however, that self-reported measures are, *per se*, more strongly correlated with the digit ratio. Rather, our results, especially Study II, show that it is important to understand the factors driving the correlations between different measures of competitiveness and the digit ratio. Study II shows that those elements of competitiveness that are not related to enjoyment of competition, e.g., the desire to perform better and to win against others, are neither significantly correlated with the behavioral measure nor with the digit ratio. Consequently, these facets of competitiveness do not seem to explain the observed patterns of correlation between different measures of competitiveness and the digit ratio. Hence, psychometric scales that do not focus on enjoyment of competition may lead to different conclusions regarding the relationship between competitiveness and digit ratios.

Follow-up studies could more comprehensively examine the different facets of competitiveness by employing behavioral measures and psychometric measures of competitiveness reflecting more facets of competitiveness. Since our findings suggest that the digit ratio is related to enjoyment of competition, we would expect that significant correlations between digit ratio and behavioral measures might be found if the latter is obtained from experimental designs that provide more opportunities for enjoyment of competition. Moreover, future research could examine the potential role of moderators for selection into competition. Moderating variables may also explain seemingly conflicting findings related to the relationship between hormones and behavior. Existing studies suggest, for instance, that interactions between hormones and contextual cues affect individuals' decisions to cooperate (e.g., Sanchez-Pages and Turiegano, 2010; Millet, 2011; Declerck et al., 2014), However, the decision to cooperate in environments characterized by elements of competition is better classified as behavior *within* competition rather than individuals' tendencies to *select into* competitive environments (Bönte et al., 2017a). Future research related to contextual cues might also more thoroughly build on demonstrated differences induced by specific cultural environments (e.g., Gneezy et al., 2009; Cárdenas et al., 2012).

Examining different behavioral and experimental measures might also be a fruitful approach for empirical studies investigating relationships between the digit ratio and other sex-dependent behaviors. For example, Brañas-Garza et al. (2017) report that their experimental measure of risk taking is significantly correlated with the digit ratios of both hands, whereas the correlation between their self-reported (single item) measure of risk taking and the digit ratio is statistically insignificant. As outlined above, the results reported by Brañas-Garza et al. also do not imply that experimental measures of risk taking are, *per se*, more strongly correlated with digit ratio than self-reported measures. Their single-item measure might be confounded by facets of risk taking that are, generally or in their specific context, not related to the digit ratio. In sum, and as already demonstrated by Bönte et al. (2017a), combining various experimental measures with different self-reported measures of competitiveness allows for a better understanding of the facets of competitiveness that are reflected by behavioral and psychometric measures and our study suggests that this approach is also useful for investigating the relation between the digit ratio and sex-dependent behaviors, like individual competitiveness.

It is a limitation of our study that we do not fully understand the causal links between digit ratio and individual competitiveness. While we discuss a potential link through prenatal testosterone exposure as well as indirect links via risk taking and confidence, there might be other sexually dimorphic behavioral traits that could be related to selection into competition or behavior in competition and that are also correlated with the digit ratio; candidates could be aggressiveness and sensation-seeking (Hampson et al., 2008). The potential causal link between competitiveness and digit ratio that we present is based on the assumption that 2D:4D is a proxy for PAE, which influences individual competitiveness through its effect on the masculinization of the brain. While the validity of 2D:4D as marker for PAE is supported by a number of studies (e.g., Manning et al., 1998; Manning, 2002; Lutchmaya et al., 2004; McIntyre et al., 2006; Hönekopp and Watson, 2010), the usefulness of 2D:4D as a proxy for PAE is also challenged in the literature. It is argued that the link between finger ratios and PAE appears too weak or absent (Hines et al., 2015; Warrington et al., 2016) and 2D:4D might be affected by other factors than PAE (cf. Medland et al., 2010; Dressler and Voracek, 2011). In any case, our results indicate that individual competitiveness is related to a sexually dimorphic biological trait, namely 2D:4D.

Another relevant limitation of our study is the measurement error that is introduced by our measurements of 2D:4D. In previous studies, numerous methods are used to measure 2D:4D and the ongoing debate about the reliability of different approaches has not yet reached consensus (e.g., Allaway et al., 2009; Ribeiro et al., 2016). We use two different measurement approaches. In Study I, the finger lengths were measured with an electronic caliper and a self-reported ruler-based measurement of 2D:4D was used in Study II. In particular, the reliability of self-measured finger lengths is an issue (Hönekopp and Watson, 2010). To address this problem, we eliminate unreliable observations by extending the measurement method of Manning and Fink (2008). Specifically, middle finger length is measured twice for each hand (once in conjunction with the index finger, then again with the ring finger), which allows us to exclude observations where the two measurements for the middle finger strongly differ. While this approach helps to increase the reliability, we still find that the standard error of the digit ratio (R2D:4D) in Study II (0.053) is somewhat higher than in Study I (0.037), while the mean value is very similar in Study I (0.991), and Study II (0.994). These potential measurement errors in our two measures tend to result in a downward (attenuation) bias of estimated effect sizes. Consequently, the estimated effect sizes of R2D:4D in both studies, and particularly in Study II, may only represent the lower bound of the true effect size.

To conclude, our study provides empirical evidence for a negative association between right-hand digit ratio (R2D:4D) and individual competitiveness, while identifying age as an important moderator. We hope that our work stimulates future research that further elaborates on the role that biological factors play for selection into competition, thereby searching for causal explanations that may guide and improve empirical research in this field.

## Ethics statement

We followed standard rules for Germany, in general, and for our university, in particular, which do not require participants' signatures, but allow an informed consent implied by behavior. That is, directly at the beginning of the survey or experiment, participants were informed in writing about the content of the survey or experiment as well as about how the data would be used, i.e., for demonstration in the following teaching within this course (for parts of the survey) and for scientific research (all data). They were explicitly informed that participation was anonymous and fully voluntary; that is they could decide to not participate and also to stop participation at any point of the survey or experiment. For Study II, which was composed of a survey and a separated experiment for a subsample, the information was repeated before they decided to participate in the experiment that accompanied the survey.

## Author contributions

WB, VP, and DU: Contributed substantially to the conception and design of the work, the analysis, and interpretation of data for the work; drafted the work and revisited it critically for important intellectual content; and approved the version to be published and agrees to be accountable for all aspects of the work. MV: Contributed substantially to the interpretation of data for the work; revisited the work critically for important intellectual content; and approves the version to be published and agrees to be accountable for all aspects of the work.

### Conflict of interest statement

The authors declare that the research was conducted in the absence of any commercial or financial relationships that could be construed as a potential conflict of interest. The reviewer LLK and handling Editor declared their shared affiliation.

## Footnotes

^1^Previous research suggests that, in particular, the digit ratio of the right hand (R2D:4D) is significantly correlated with sex-dependent behavioral traits (Fink et al., 2004; Hampson et al., 2008).

^2^While HOX genes have a fundamental role in embryonic development, with the differentiation of fingers and toes influenced by HOXA and HOXD genes (Manning et al., 2003), in recent genome-wide association studies (GWAS) of 2D:4D no signal emerged that HOX genes would impact 2D:4D (Medland et al., 2010; Lawrance-Owen et al., 2013; Warrington et al., 2016).

^3^The conceptualization of competitiveness as tendency to self-select into competitive environments should be distinguished from three alternative conceptualizations. First, it differs from individuals' responses within a competitive environment (Croson and Gneezy, 2009; Bönte et al., 2017a). For example, willingness to win might trigger individuals to increase their efforts to leverage odds of winning in response to being in a competitive environment, independent of whether or not they seek competitive environments. It also differs from individuals' tendencies to maximize own, relative to others', rewards. While individuals maximizing relative rewards are sometimes considered to be competitive individuals (e.g., van Lange et al., 1997; Fehr and Schmidt, 1999), this defining feature does not relate to the selection into competitive situations, but rather to behavior within competitive environments. Last, we distinguish individual competitiveness as selection into competitive environments from competitiveness as ability to win (physical) competitions or as (physically) best performing (e.g., Manning and Taylor, 2001; Hönekopp et al., 2006). While individuals who believe they will be more likely to win might also be more likely to enter competitions, this would not reflect a unique preference for competition, but only a preference to maximize one's expectancies. Thus it is not only expectations about winning but also individuals' willingness to take risks that might make individuals look as if they favor competitive environments (Gneezy et al., 2003). Consistent with previous research, we distinguish such beliefs and preferences, which may make individuals look like being competitive, from individual competitive preferences.

^4^The gender difference in tournament entry in stereotypical male tasks persists after controlling for performance, confidence, and risk attitudes (Niederle, 2016). The gender gap tends to be reduced or vanishes if tasks are not male stereotyped and time constraints are removed (Shurchkov, 2012).

^5^There is some, but not yet replicated, evidence from a lab-in-the-field experiment conducted by Mayr et al. (2012) that competitiveness of both men and women changes with age and, specifically, displays an inverse U-shaped relationship. Moreover, Mayr et al. (2012) show that age does not notably affect the difference between genders in competitiveness throughout the life span. Using a representative data set of more than 25,000 individuals from 36 countries and a self-reported measure of competitiveness, Bönte (2015) confirms this finding, reporting that gender differences among adult men and women are hardly affected by age. It is also demonstrated that gender differences in competitiveness already exists at a young age in experimental studies focusing on samples consisting of children (Gneezy and Rustichini, 2004; Sutter and Rützler, 2010).

^6^Empirically supporting this view, van Honk et al. (2012) demonstrate that the negative effect of testosterone administration on cognitive empathy in the context of human bargaining behavior is boosted by high levels of PTE.

^7^Methodological differences did not affect the behavioral measure of competitiveness: not the experimenter's gender [χ2_(1)_ = 0.28, *p* = 0.60], not the day of the experiment [χ2_(5)_ = 1.71, *p* = 0.89], not the type of another game they were exposed to [χ2_(2)_ = 0.92, *p* = 0.63], and not whether the measurement was taken before or after this other game [χ2_(1)_ = 0.90, *p* = 0.34].

^8^The two common methods used in previous research to measure digit ratio 2D:4D are the direct and the indirect approaches. While direct approach measures finger length directly on the finger, the indirect approach is based on indirectly measured fingers from photocopies or scans. Hence, we had to choose between indirect and direct measurement of finger lengths. We opted for a direct measurement of the digit ratio presuming that visitors of a shopping mall are likely to be suspicious of scanning their entire hands.

^9^While Appendix C could be interpreted as perhaps indicating an inverse U-shaped moderating effect of age on the link between 2D:4D and self-reported competitiveness, none of further tests of such effects are statistically significant.

^10^Behavioral measure of competitiveness (two-sample test of proportions: *z* = 0.38, *p* = 0.70), the two self-reported measures of individual competitiveness (HS: *t* = 0.26, *p* = 0.79; EC: *t* = 0.62, *p* = 0.54), and the right- and left-hand second to fourth digit ratio (right: *t* = 0.29, *p* = 0.77; left: *t* = 1. 24, *p* = 0.21).

^11^As the whole study was conducted in class, all participants knew their potential competitors. The matching pool of competitors included only those participants who selected the tournament. Participants were not provided any information regarding the matched competitor.

^12^Note that when considering the descriptive statistics reported in Tables 1-I, II, we see that despite the sample means of measures of right-hand and left-hand 2D:4D are of comparable sizes in Studies I and II, the standard errors are substantially larger in Study II, which is based on the self-reported measure of 2D:4D. This observation could indicate that this measure is subject to larger measurement errors.

^13^Appendix C reports analyses for further splitting the group of those older than 25 years.
