# Supplementary material for: Digit Ratio (2D:4D) Predicts Self-Reported Measures of General Competitiveness, but Not Behavior in Economic Experiments
Source: Front Behav Neurosci. 2017 Dec 8;11:238. doi: 10.3389/fnbeh.2017.00238 (PMC5728070; doi:10.3389/fnbeh.2017.00238)
Supplement: Supplementary file 1 [file Appendix.pdf]

APPENDIX A
Analysis of gender differences in the association of R2D4D and competitiveness

Our estimation results suggest that the estimated effect of R2D:4D does not differ in a statistically significant way between men and women, neither for the behavioral nor for the self-reported measure of competitiveness. Note, equivalent analyses for the left hand (available upon request) indicate that in all these samples, this difference is also not significant for the left hands. Furthermore, especially for the relevant right-hand ratio, R2D:4D, the directions of these gender differences differ between the two samples. We find that although the negative relationship between R2D:4D and self-reported competitiveness is less negative for females in the general sample, it is more negative for them in the student sample. Effectively, in the general sample the effect of females is not statistically significant for females (females: β=-1.640, S.E.= 2.798, p=0.558; males: β=-6.493, S.E.= 2.856, p=0.024), but in the student sample the effect is not statistically significant for males (females: β=-3.336, S.E.= 1.304, p=0.005; males: β=-0.861, S.E.= 1.706, p=0.614). For the young general sample, the effect is statistically significant for both females and males (females: β=-9.860, S.E.= 4.942, p=0.049; males: β=-10.331, S.E.= 5.953, p=0.086). Thus, in contrast to the overall rather robust and consistent pattern for the association between R2D:4D and competitiveness, the observed pattern related to a possible gender-specificity of this relationship is ambiguous and not consistent between the samples. Future research and meta-analyses employing even larger samples might shed further light on the potential gender-specificity of this the relationship between R2D:4D and competitiveness.

	**Behavioral measurement (BM)**			**Self-reported measurement (EC)**		
	**Study I**	**Study I**	**Study II**	**Study I**	**Study I**	**Study II**
Population	*General*	*General ≤ 25y*	*Student ≤ 25y*	*General*	*General ≤ 25y*	*Student ≤ 25y*
R2D:4D	0.273	1.560	2.492	-4.067*	-10.095*	-2.263*
	(2.725)	(5.589)	(4.060)	(1.999)	(3.869)	(1.074)
Gender contrast	-0.543	2.097	-2.646	-2.798	-0.426	0.986
	(2.703)	(5.530)	(4.018)	(1.983)	(3.833)	(1.064)
R2D:4D × Gender contrast	0.227	-2.443	1.696	2.427	0.235	-1.402
	(2.725)	(5.589)	(4.060)	(1.999)	(3.869)	(1.074)
Constant	-0.137	-1.150	-3.168	8.390***	14.534***	6.780***
	(2.703)	(5.530)	(4.018)	(1.983)	(3.833)	(1.064)
Observations	418	108	131	418	108	581
Fit index LL / R^2	-283.89*	-72.00	-70.21***	0.082***	0.090*	0.110***
Fit statistic (χ^2 / F)	(10.32)	(2.71)	(22.40)	(12.40)	(3.42)	(23.86)

Notes: R2D:4D = 2D:4D of right hand. Table reports estimated coefficients and standard errors (in parentheses). Samples exclude left-handed participants and, for Study 1, we also provide an analysis of the subsample of younger people, which both focuses on those individuals assumed to display stronger relationships between R2D:4D and competitiveness. The gender difference is operationalized as contrast code (-1 for males and +1 for females), such that the coefficient of the R2D:4D reflect the average of the effects for females and males. The coefficient of the contrast code indicates the extent to which females and males score above respectively below this average. Significance levels: + p<0.10, * p<0.05, ** p<0.01, ***p<0.001

APPENDIX B
Competitiveness items, their sources and how they are assigned to measures in this study

No.	Source	Measure	Items	
1	HS	HS, EC	English	I enjoy working in situations involving competition with others.
			Replaced	I like situations in which I compete with others.
			German	Ich mag Situationen, in denen ich mit anderen konkurriere.
2	HS	HS	English	It annoys me when other people perform better than I do.
			German	Es ärgert mich, wenn andere bessere Leistungen bringen als ich.
3	HS	HS	English	It is important to me to perform better than others on a task.
			German	Es ist für mich wichtig, eine Aufgabe besser zu erfüllen als andere.
4	HS	HS	English	I feel that winning is important in both work and games.
			German	Sowohl im Beruf als auch im Spiel möchte ich gewinnen.
5	HS	HS	English	I try harder when I'm in competition with other people.
			German	Ich gebe mir mehr Mühe, wenn ich mit anderen Menschen im Wettbewerb stehe.
6	NK	EC	English	I enjoy competing against others.
			German	Mit anderen zu wetteifern, macht mir Spaß.
7	SH	EC	English	I find competitive situations unpleasant.
			German	Ich empfinde Wettbewerbssituationen als unangenehm.

Notes: HS = Helmreich and Spence (1978); NK = Newby and Klein (2014); SH = Smither and Houston (1992).

APPENDIX C

Further exploring the moderating effect of age: Regressions for all age quartiles.

	Behavioral measure (logistic regression)				Self-reported measure (ordinary least squares regression)			
**Model**	**1**	**2**	**3**	4	**5**	**6**	**7**	8
Subsample (age quartiles)	1st	2nd	3rd	4th	1st	2nd	3rd	4th
R2D:4D	1.492	-0.990	1.516	-6.300	-9.979**	4.948	-3.206	-5.293
	(5.420)	(6.161)	(5.453)	(5.794)	(3.476)	(3.678)	(3.582)	(3.688)
Risk taking	0.205	0.256+	-0.061	0.194	0.226*	0.279**	0.383***	0.452***
	(0.152)	(0.149)	(0.137)	(0.151)	(0.095)	(0.089)	(0.093)	(0.097)
Conf.: Wining Prob.	0.397**	0.478***	0.545***	0.245+	0.016	0.090	-0.011	0.139+
	(0.135)	(0.141)	(0.156)	(0.125)	(0.076)	(0.081)	(0.086)	(0.076)
Female	-0.459	-0.466	0.793+	-0.731+	-0.435	-0.865**	-0.227	-0.464
	(0.421)	(0.434)	(0.442)	(0.428)	(0.269)	(0.279)	(0.285)	(0.283)
Constant	-3.946	-2.482	-4.582	4.695	13.463***	-2.143	5.875	7.088+
	(5.536)	(6.116)	(5.450)	(5.904)	(3.494)	(3.660)	(3.568)	(3.756)
Observations	120	115	117	109	120	115	117	109
Fit index LL / R^2	-73.24**	-69.22***	-72.90**	-67.90*	0.147**	0.207***	0.147**	0.288***
Fit statistic (χ^2 / F)	(17.73)	(20.98)	(16.18)	(13.24)	(4.95)	(7.17)	(4.81)	(10.51)

Notes. R(L)2D:4D = 2D:4D of right (left) hand. Table reports estimated coefficients and standard errors (in parentheses). Ranges for age quartiles: 1st: ≤25 years, 2nd: 26-36 years, 3rd: 37-50 years, 4th: >50 years.
Significance levels: + p<0.10, * p<0.05, ** p<0.01, ***p<0.001
